# Characterising *Pv*RBSA: an exclusive protein from *Plasmodium* species infecting reticulocytes

**DOI:** 10.1186/s13071-017-2185-6

**Published:** 2017-05-18

**Authors:** Darwin A. Moreno-Pérez, Luis A. Baquero, Diana M. Chitiva-Ardila, Manuel A. Patarroyo

**Affiliations:** 1 Molecular Biology and Immunology Department, Fundación Instituto de Inmunología de Colombia (FIDIC), Carrera 50 No. 26-20, Bogotá, D.C. Colombia; 2 Programme in Biomedical and Biological Sciences, Universidad del Rosario, Carrera 24 No. 63C-69, Bogotá, D.C. Colombia; 3 Basic Sciences Department, School of Medicine and Health Sciences, Universidad del Rosario, Carrera 24 No. 63C-69, Bogotá, D.C. Colombia

**Keywords:** *Plasmodium vivax*, Antigenic protein, Adhesin, Reticulocyte

## Abstract

**Background:**

*Plasmodium vivax* uses multiple ligand-receptor interactions for preferential invasion of human reticulocytes. Several of these ligands have been identified by *in silico* approaches based on the role displayed by their orthologs in other *Plasmodium* species during initial adhesion or invasion. However, the cell adhesion role of proteins that are exclusive to species that specifically invade reticulocytes (as *P. vivax* and *P. cynomolgi*) has not been evaluated to date. This study aimed to characterise an antigen shared between *Plasmodium* species that preferentially infect reticulocytes with a focus on assessing its binding activity to target cells.

**Results:**

An *in silico* analysis was performed using *P. vivax* proteome data to identify and characterise one antigen shared between *P. vivax* and *P. cynomolgi*. This led to identification of the *pvrbsa* gene present in the *P. vivax* VCG-I strain genome. This gene is transcribed in mature schizonts and encodes a protein located on the parasite surface. r*Pv*RBSA was antigenic and capable of binding to a population of reticulocytes with a different Duffy phenotype. Interestingly, the molecule showed a higher percentage of binding to immature human reticulocytes (CD71^hi^).

**Conclusions:**

This study describes for the first time, a molecule involved in host cell binding that is exclusive in reticulocyte-infecting *Plasmodium* species. This suggest that *Pv*RBSA is an antigenic adhesin that plays a role in parasite binding to target cells.

**Electronic supplementary material:**

The online version of this article (doi:10.1186/s13071-017-2185-6) contains supplementary material, which is available to authorized users.

## Background

Basic research in *P. vivax* has been delayed, mainly due to difficulties associated with its in vitro propagation, resulting from the predilection of this species for invading immature erythrocyte cells (reticulocytes) [1, 2]. Consequently, bioinformatics approaches represent a good solution for identifying *in silico* vaccine candidates in *P. vivax* by comparative analysis, bearing in mind that many invasion-associated proteins from other *Plasmodium* species have already been described. Information derived from omics studies of *P. vivax* (genome [3], transcriptome [4] and proteome [5–8]) has been useful for large-scale analysis of gene composition, transcripts and parasite proteins and, importantly, facilitate *in silico* predictions on the function of many *P. vivax* proteins.

Furthermore, *in silico* tools have been instrumental in characterising some *P. vivax* molecules interacting with reticulocytes, such as the Duffy binding protein (DBP) [9], reticulocyte binding proteins (RBP) [10–12], merozoite surface protein-1 (MSP-1) [13], rhoptry neck protein-5 (RON5) [14] and, recently, the *P. vivax* GPI-anchored micronemal antigen (GAMA) protein (manuscript in press). However, the number of *P. vivax* target cell binding proteins identified to date is low compared to available information on *P. falciparum*, suggesting that further studies are required to supplement the current set of *P. vivax* adhesin data, to improve our understanding of the molecular basis of parasite invasion.

Identifying *P. vivax* molecules with a role in host cell invasion by their similarity with proteins in *P. falciparum* has been a very promising approach. However, this has limitations when identifying those molecules involved in parasite recognition and invasion of reticulocytes. This study aimed to characterise a specific molecule from species infecting reticulocytes (e.g. *P. vivax* and *P. cynomolgi*) by determining its target cell binding profile.

## Methods

### Bioinformatics analysis, primer design and peptide synthesis

The currently available information published in *P. vivax* proteome studies [5–8] was used as the source for analysing *in silico* proteins which might be vaccine candidates. The criteria for selecting proteins included: a prominent expression of the codifying genes > 35 h post-invasion (required) according to transcriptome study of the *P. vivax* intra-erythrocyte life-cycle [4]; a positive prediction by SignalP 4.1 [15] and BaCelLo [16] of a secretion signal sequence and extracellular localisation, respectively; the presence (or not) of a GPI anchor sequence using FragAnchor software [17], as well as the presence of repeats having 90% similarity in amino acid (aa) sequences using T-REKS algorithm [18]. The Phobius [19], HMMTOP [20] and TMHMM [21] servers were used to predict transmembrane regions. The selected genes were analysed to identify orthologs in other *Plasmodium* species according to the PlasmoDB [22] and the Kyoto Encyclopedia of Genes and Genomes ortholog clusters (KEGG OC) [23] databases. The sequence of any gene selected for being characterised was scanned in the PlasmoDB database and used for manually designing specific primers (using Generunner software, version 3.05), the same as for B-cell linear epitopes all along their encoding sequence, predicting the highest average values for hydrophilicity, solvent accessibility and Parker’s antigenicity using ANTHEPROT software [24].

### Propagating VCG-I strain parasites and isolating schizonts

Vivax Colombia Guaviare-I (VCG-I) strain parasites were propagated six years ago and used as the source of biologic material, as previously described in detail [25]. The blood sample containing parasite-infected cells was collected in heparin tubes and passed through a discontinuous Percoll gradient (GE Healthcare, Uppsala, Sweden), according to an already-established protocol [26]. The schizont-stage enriched parasites were isolated from cells by incubating them for 5 min in 0.02 mM saponin buffer containing 7 mM K_2_HPO_4_, 1 mM NaH_2_PO_4_, 11 mM NaHCO_3_, 58 mM KCl, 56 mM NaCl, 1 mM MgCl_2_ and 14 mM glucose, pH 7.5 and then washed extensively with PBS, pH 7.0.

### Extracting biological material

Isolated parasites were used as RNA, genomic DNA (gDNA) and total protein source. Total RNA was extracted from the sample using the Trizol method and treated with *RQ1* (RNA-qualified) RNase-free DNase (Promega, Madison, USA) according to the manufacturer’s recommendations. SuperScript III enzyme (RT+) (Invitrogen, Carlsbad, USA) was used for synthesising complementary DNA (cDNA) in the following conditions: 65 °C for 5 min, 50 °C for 1 h and 70 °C for 15 min. An additional reaction without the SuperScript III enzyme (RT-) was used as negative control, following 15 min incubation at 37 °C with RNase (Promega). A Wizard Genomic purification kit (Promega) was used for obtaining the gDNA. Regarding protein extraction, the parasites were homogenised in lysis buffer containing 5% SDS, 10 mM PMSF, 10 mM iodoacetamide, 1 mM EDTA and then spun at 16,000× *g* for 5 min. The proteins were recovered from the supernatant and quantified using a BCA protein assay kit (Thermo Scientific, Rockford, USA). RNA, cDNA, gDNA and total protein were stored at -70 °C until later use.

### Gene cloning and sequencing

The gDNA and cDNA (RT+ and RT-) samples were used as template in 25 μl PCR reactions containing 1× KAPA HiFi HotStart ReadyMix (KAPA Biosystems, Woburn, MA, USA), 0.3 μM primers and DNAse-free water for completing the reaction volume. Specific primers were designed for amplifying the entire *P. vivax reticulocyte binding surface antigen* (*pvrbsa*) gene (Forward 5'-ATG AAA GGA ATA ATG AAT GG TT-3' and Reverse 5'-ATA ACC ATC CAA ATC GTC AAA-3') or for producing the recombinant protein excluding the signal peptide and the transmembrane region (Forward 5'-ATG ATA TTG TAC AGC GAC GAC TC-3' and Reverse 5'-GCT ATC TTT CTT CAC ATT ATA C-3').

The PCR began with a denaturing step at 98 °C for 3 min, followed by 35 cycles at 98 °C for 20 s, 56 °C for 15 s and 72 °C for 30 s. A Wizard PCR preps kit (Promega) was used for purifying gene amplicons obtained from three independent PCRs done with the RT+ and gDNA samples, once quality had been evaluated on agarose gel. Purified products were ligated to the pEXP5 CT/TOPO expression vector or in a new *in house* designed vector (pELMO) [27] for the gene obtained from gDNA and transformed in *E. coli* TOP10 chemically competent cells (Invitrogen). Several clones were grown for purifying the plasmid using an UltraClean mini plasmid prep purification kit (MO BIO Laboratories, California, USA). The insert integrity and correct orientation were then confirmed by sequencing, using an ABI-3730 XL sequencer (MACROGEN, Seoul, South Korea). ClustalW (NPS) software [28] was used for comparing manually the gene sequences from the Sal-I reference strain [3] and the primate-adapted VCG-I strain.

### Recombinant protein expression and extraction

*E. coli* BL21-DE3 (Invitrogen) cells which had been previously transformed with the recombinant plasmids were grown in Luria-Bertani (LB) medium containing 100 μg/ml ampicillin, overnight at 37 °C using a Lab-line Incubator Shaker. The initial inoculum was seeded in 1 l LB and handled in the aforementioned conditions until reaching 0.5 OD_600_. After the culture was incubated on ice for 30 min, IPTG 1 mM was then used to induce expression for 16 h at room temperature (RT) at ~200 rpm. The cells were harvested by spinning at 2,400× *g* for 20 min and used for native extraction procedures. A new protocol for extracting proteins in a soluble form was used. Briefly, cellular pellet obtained from *E. coli* expressing *Pv*RBSA was freeze/thawed for 3 cycles and then homogenised in native extraction buffer (NEB) (50 mM Tris-Cl, 300 mM NaCl, 25 mM imidazole, 0,1 mM EGTA and 0.25% Tween-20, pH 8.0). The mixture was then incubated for 1 h at 4 °C at 10 rpm using a tube rotator (Fisher Scientific, Waltham, USA) and the supernatant was collected by spinning at 16,000× *g* for 1 h.

### Protein purification

Solid-phase affinity chromatography was used for protein purification. The Ni^+2^-NTA resin (Qiagen, Valencia, CA, USA) was pre-equilibrated with NEB buffer, incubated with *E. coli* lysate overnight at 4 °C and the protein-resin mixture was then placed on a column. The unbound proteins were eluted by washing with 20 ml NEB buffer containing 0.1% Triton X-114 followed by 50 ml of the same buffer without detergent. Bound proteins were eluted with PBS containing imidazole at increasing concentrations (50 mM to 500 mM) in 3 ml fractions. The purification was confirmed by Coomassie blue staining and the fractions pooled and dialysed extensively in PBS, pH 7.2. The protein was quantified using a micro BCA protein assay kit (Thermo Scientific) and bovine serum albumin (BSA) as reference curve.

### Obtaining polyclonal antibodies

The VCG-I strain *Pv*RBSA sequence was used for designing two 20 aa-long peptides (CG-KRNSSVSSLDSDMGSYKNKS-GC (peptide 39478) and CG-VFGKGRKKPMKVKKGGGKIS-GC (peptide 39480)) which were then synthesised, according to a previously-established methodology [29], polymerised, lyophilised and characterised by RP-HPLC and MALDI-TOF MS. New Zealand rabbits were immunised with a 500 μg dose of each synthetic peptide emulsified in Freund’s complete adjuvant (FCA) (Sigma, Missouri, USA) on day 0, whilst the same emulsified mixture in Freund’s incomplete adjuvant (FIA) was inoculated on days 21 and 42. The pre-immune sera were collected before the first immunisation and hyper-immune sera were collected 20 days after the last dose. Specific antibodies were purified by affinity chromatography using CNBr-activated Sepharose 4B (Amersham, Uppsala, Sweden). Briefly, 5 μmol of peptide were diluted in coupling buffer (0.1 M NaHCO_3_, 0.5 M NaCl pH 8.3) and then incubated for 16 h at 4 °C with Sepharose resin. After washing ligand excess with 5 volumes of coupling buffer, the resin-free groups were blocked with 0.1 M buffer Tris–HCl for 2 h at quiescence at RT, followed by washing the resin 3 times with alternate pH solutions (0.1 M acetate buffer with 0.5 M NaCl, pH 4.0 and 0.1 M Tris-HCl with 0.5 M NaCl, pH 8.0). Five ml of each rabbit hyper-immune serum (diluted at 1:1 ratio with buffer coupling) were passed through the resin after being homogenised with PBS. Unbound antibodies were washed with 10 ml buffer coupling while strongly bound antibodies were eluted with 1 ml elution buffer (0.1 M glycine pH 7, 6, 5, 3.9 and 2.9) at descendant pH and neutralised with 1 M Tris pH 8.0 in a 1:9 ratio (elution buffer:neutralisation buffer). The antibodies were incubated with 45% ammonium sulphate for 1 h on ice with constant stirring and then for 16 h at 4 °C without shaking. After spinning at 16,000× *g* for 15 min, the pellet was homogenised in 100 μl PBS and the sample was extensively dialysed and stored at -20 °C until use.

### Protein localisation by indirect immunofluorescence (IFI)

Slides containing *Aotus* monkey infected reticulocytes were previously prepared, as described in previous work [30]. The samples were fixed and permeabilised by incubating them for 5 min at RT with PBS containing 4% paraformaldehyde (v/v) and then with PBS with 0.1% Triton X-100 (v/v). After blocking with 1% BSA-PBS solution (*v/v*) for 1 h at RT, each sample was incubated with anti-*Pv*RBSA rabbit antibodies (1:30) or anti-*Pv*RON2 mouse antibodies (1:20) in the same conditions. FITC-conjugated anti-rabbit IgG antibody (Sigma) at 1:30 dilution and Rhodamine-conjugated anti-mouse IgG antibody (1:200) monoclonal secondary antibodies were used for 1 h in darkness at RT. DAPI (0.5 μg/ml) was used for staining parasite nuclei for 10 min at RT and then was washed several times with PBS to remove excess reagent. The slides were examined under a florescence microscope (Olympus BX51) using 100× oil immersion objective.

### Western blot analysis of recombinant and parasite proteins

Total parasite and recombinant proteins were separated on 12% SDS-PAGE and transferred to nitrocellulose membranes which were blocked with 5% skimmed milk in TBS-0.05% Tween for 1 h. The membrane was cut into strips to be incubated for 1 h at RT with rabbit anti-*Pv*RBSA purified antibodies (1:100 dilution) and then with the phosphatase-coupled goat anti-rabbit IgG monoclonal secondary antibody (1:5,000) (Catalogue 9503 F, ICN) in the same conditions. The positive control for r*Pv*RBSA Western blotting was a strip incubated with peroxidase-coupled mouse anti-histidine monoclonal antibody (1:4,500) (Catalogue A7058, Sigma). The blots were revealed with a BCIP/NBT colour development substrate kit (Promega) or VIP peroxidase substrate kit (Vector Laboratories, Burlingame, Canada) according to the manufacturers’ indications. Each band’s expected weight was determined by linear regression using XL-OptiProtein (Applied Biological Materials Inc, Richmond, BC, Canada) weight marker as reference.

### Enzyme-linked immunosorbent assay (ELISA)

The recombinant protein was used for evaluating the presence of anti-r*Pv*RBSA antibodies in samples taken from *P. vivax-*exposed individuals (who had suffered at least one episode of infection) in the municipality of Tierra Alta, Córdoba. The negative controls used here came from sera from healthy individuals who had never been affected by the disease. The ELISA was performed as described previously [30].

### Cell binding assay

Cord blood samples were typified for determining the Duffy phenotype (Fya^+^/Fyb^−^; Fya^−^/Fyb^+^; Fya^+^/Fyb^+^) by standard blood banking methods using anti-Fya and Fyb sera. Five μL of cells were then incubated with 25 μg r*Pv*RBSA for 16 h at 4 °C at 4 rpm. DBP region II and III/IV were used as positive and negative controls, respectively [9]. After washing with 1% BSA-PBS solution (v/v), the sample was incubated with mouse anti-His-PE monoclonal antibody (1:40 dilution) (MACSmolecular-Miltenyi Biotec, San Diego, CA, USA) for 30 min in darkness. Reticulocytes and white cells were stained by incubating with anti-CD71 APC-H7 Clone M-A712 (1:80 dilution) (Becton Dickinson, Franklin Lakes, NJ, USA) and anti-CD45 APC clone 2D1 (1:80 dilution) (Becton Dickinson) monoclonal antibodies for 20 min at RT. A FACSCanto II cytometer (BD, San Diego, CA, USA) was then used for quantifying erythrocyte binding and FlowjoV10 software for analysing 1 million events. PE signal intensity was evaluated as a function of CD71 signal to determine CD71 low (CD71^lo^) and high (CD71^hi^) cells.

### Statistical analysis

Statistical significance was assessed by comparing means, using a 0.05 significance level. Mann-Whitney U-test analysis was used for comparing the mean of the experimental group with the control in ELISA. Differences between means were compared by Tukey’s range test when comparing multiple groups or *t*-test for comparing two groups for binding assays. GradhPad Software (San Diego, CA) was used for all statistical analysis. Mean values and standard deviations (SD) were calculated from the measurements of three independent experiments.

## Results

### Predicting *P. vivax* invasion-related proteins

The criteria established in the methodology led to identifying several genes encoding *P. vivax* molecules which play a role in cell binding (as previously reported), such as the RBPs [31], some RONs [14] and GAMA. Interestingly, one gene encoding a 48 kDa protein (PlasmoDB database ID: PVX_096055) was identified which, apart from *P. vivax*, was also present in *Plasmodium cynomolgi* (one species infecting reticulocytes). This gene was named the *P. vivax* reticulocyte binding surface antigen (*Pv*RBSA) according to the results showed in this study.

**Fig. 1:**
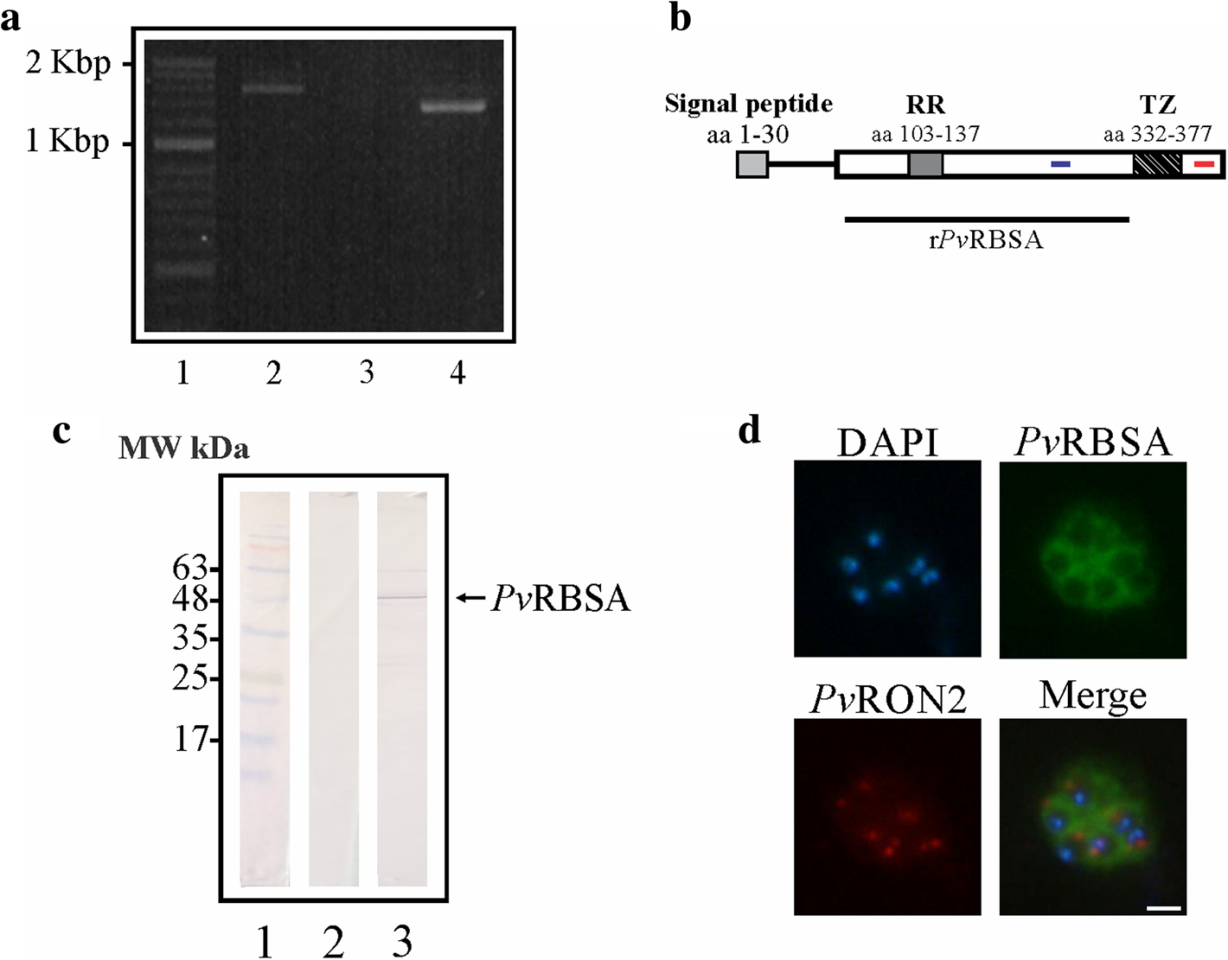
*Pv*RBSA characterisation in the *P. vivax* VCG-I strain. **a** *pvrbsa* gene presence and transcription in schizonts. Lane 1 indicates the molecular weight pattern. Lanes 2 to 4 show gene amplification using gDNA, cDNA-RT- and cDNA-RT+, respectively. **b** *In silico* characterisation of *Pv*RBSA. The diagram shows signal peptide, repeat region (RR) and transmembrane zone (TZ) location. The peptides selected for rabbit immunisation are highlighted in *blue* (peptide 39478) and *red* (peptide 39480) lines. The recombinant protein region expressed is shown by a *black line*. **c** Recognition of *Pv*RBSA in parasite lysate. Lane 1 indicates the molecular weight (MW) marker. Lanes 2 and 3 show recognition of the protein using pre-immune and hyper-immune sera, respectively. **d** Sub-cellular location of *Pv*RBSA in schizonts. The images show recognition of *Pv*RBSA (*green*), *Pv*RON2 (*red*) and the nuclei (*blue*). *Scale-bar*: 1 μm

Regarding the *pvrbsa* gene, it presence and transcription in the *P. vivax* VCG-I strain was confirmed by PCR using specific primers (designed using the Sal-I strain gene sequence) and schizont gDNA and cDNA as template. Fig. 1a shows a 1.4 to 1.6 kbp amplification product using gDNA (Lane 2) corresponding to the complete gene whilst a 1.2 to 1.4 kbp product was obtained using cDNA as template (Lane 4). No product was amplified in the control sample, thereby indicating that the synthesised cDNA had not become contaminated with gDNA (Fig.1a, Lane 3). Aligning the gene sequences from the *Aotus* monkey- adapted VCG-I strain with those from the Sal-I reference strain led to one synonymous, 9 non-synonymous mutations and one deletion being identified (Table 1). Comparing the sequences obtained from cDNA (1,269 bp) (deposited in the NCBI under GenBank access KY349105) and gDNA (1,485 bp) led to observing that the *pvrbsa* gene was encoded by two exons, the first covering the signal peptide according to *in silico* prediction (D-cutoff = 0.450) (Fig. 1b). The *pvrbsa* gene encoded a 423 aa long protein having a molecular weight of around ~47.09 kDa including a signal peptide and being 7 residues shorter than that for the Sal-I strain. *Pv*RBSA had 2 transmembrane regions located between residues 332 to 377 according to prediction by TMHMM, Phobius and HMMTOP servers and one repeat region (RR) located in aa 103 to 137, consisting of residues LT(G/E)S(N/R)ES as predicted by T-REKS (Fig. 1b); these have been numbered according to the VCG-I strain *Pv*RBSA amino acid sequence. Amplifying *pvrbsa* from the synthesised cDNA sample confirmed that the gene was transcribed in schizonts, coinciding with transcriptional analysis of 3 *P. vivax* clinical isolates where *pvrbsa* had a prominent expression profile during TP7-TP9 times corresponding to parasite development during the mature stage (early and late schizonts) of the intra-reticulocyte life-cycle [4].

**Table 1:** *Pv*RBSA mutations found by comparing the nucleotide and amino acid sequences of *P. vivax* VCG-I and Sal-1 strains

Changes in *Pv*RBSA nucleotide sequence in Sal-I and VCG-I *P. vivax* strains^a^	Changes in *Pv*RBSA aa sequences in Sal-I and VCG-I *P. vivax* strains^a^	Mutation
c.29A > G	p.Tyr10Cys	Non-synonymous
c.391_411delCTAACAGGAAGTAATGAATCC	p.Leu131_Ser137del^b^	Deletion
c.533 T > G	p.Phe178Cys	Non-synonymous
c.796A > G	p.Lys266Gly	Non-synonymous
c.797A > G	p.Lys266Gly	Non-synonymous
c.801 T > A	p.Ser267Arg	Non-synonymous
c.803A > C	p.Tyr268Ser	Non-synonymous
c.808C > G	p.His270Asp	Non-synonymous
c.845C > T	p.Pro282Leu	Non-synonymous
c.1029 T > C	p.Lys343Lys	Synonymous
c.1091G > C	p.Trp364Ser	Non-synonymous

^a^Nucleotide and amino acid positions are numbered according to the Sal-I reference strain sequence alignment with the VCG-I strain

^b^Relative location for a region having 4 identical tandem repeats from amino acid position 103 to 130 in the VCG-I strain

### *Pv*RBSA characterisation by molecular biology tools

Antibodies directed against 39478 and 39480 synthetic peptides were purified and used for evaluating the protein’s presence and location in mature parasite forms (schizonts). Specific anti-*Pv*RBSA antibodies detected one band in *P. vivax* VCG-I strain lysate treated in reduced conditions above the expected size by *in silico* analysis (43.8 kDa without the signal peptide) (Fig. 1c). Such discrepancy can be explained by anomalous migration caused by several acidic residues in the protein sequence (aspartic and glutamic acids). The antibodies also led to a surface fluorescence signal being visualised in mature schizonts like a “bunch of grapes”, this being characteristic of proteins expressed on merozoite surface (Fig. 1d). There was no signal overlap for one apical marker (*Pv*RON2). These findings led to suggesting that the *pvrbsa* transcript gave a protein product in *P. vivax* VCG-I strain schizonts, as shown in an earlier study by mass spectrometry analysis [6].

**Fig. 2:**
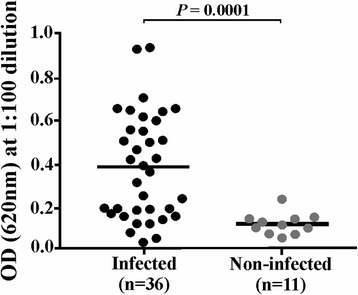
Antigenicity assay. The dot plot shows OD distribution (*Y-axis*) for detecting the *Pv*RBSA in infected and non-infected patients’ sera (X-axis). A statistically significant difference between groups was observed (Mann-Whitney *U*-test: *U* = 52, *Z* = -3.66, *P* = 0.0001)

According to the classic approach, antigenic proteins should be considered for vaccine development given that a response against them could inhibit interaction with cells. Hence r*Pv*RBSA was expressed, purified and successfully obtained in soluble form (Additional file 1: Figure S1) to evaluate its antigenicity using sera from patients suffering *P. vivax* malaria and sera from people who had never suffered the disease. The screening gave 61% seropositivity in the patients group. The statistical test gave a significant difference between the means for recognition by the sera from the infected patients group (X̄ ± SD = 0.38 ± 0.24) and the control group (X̄ ± SD = 0.12 ± 0.05) (Mann-Whitney U-test: U = 52, *Z* = -3.66, *P* = 0.0001) (Fig. 2), thereby highlighting that the protein was able to induce an immune response during natural infection.

### *Pv*RBSA interaction with human reticulocytes

**Fig. 3:**
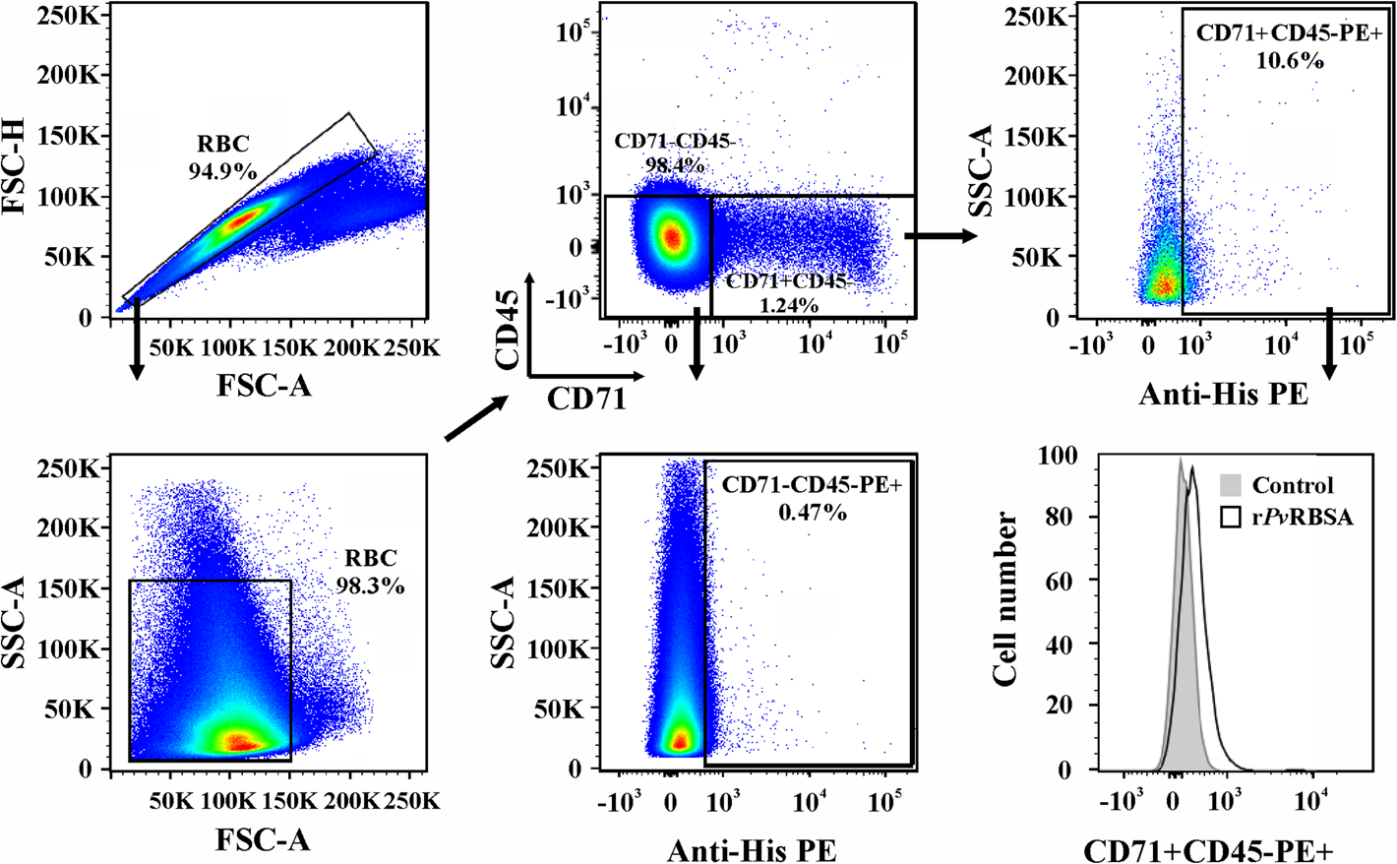
Gating strategy for selecting reticulocytes and mature erythrocyte cell population. Doublets were excluded comparing FSC-H to FSC-A cytogram. Cells were then selected by their granularity by plotting SSC-A against FSC-A. The CD45 and CD71 signals were plotted for selecting reticulocyte (CD71 + CD45-) and mature erythrocyte (CD71-CD45-) populations. The cell percentage having bound protein was calculated using the PE signal against SSC-A. A representative histogram from three independent experiments analysing the PE signal for the r*Pv*RBSA binding assay compared with the control is shown

**Fig. 4:**
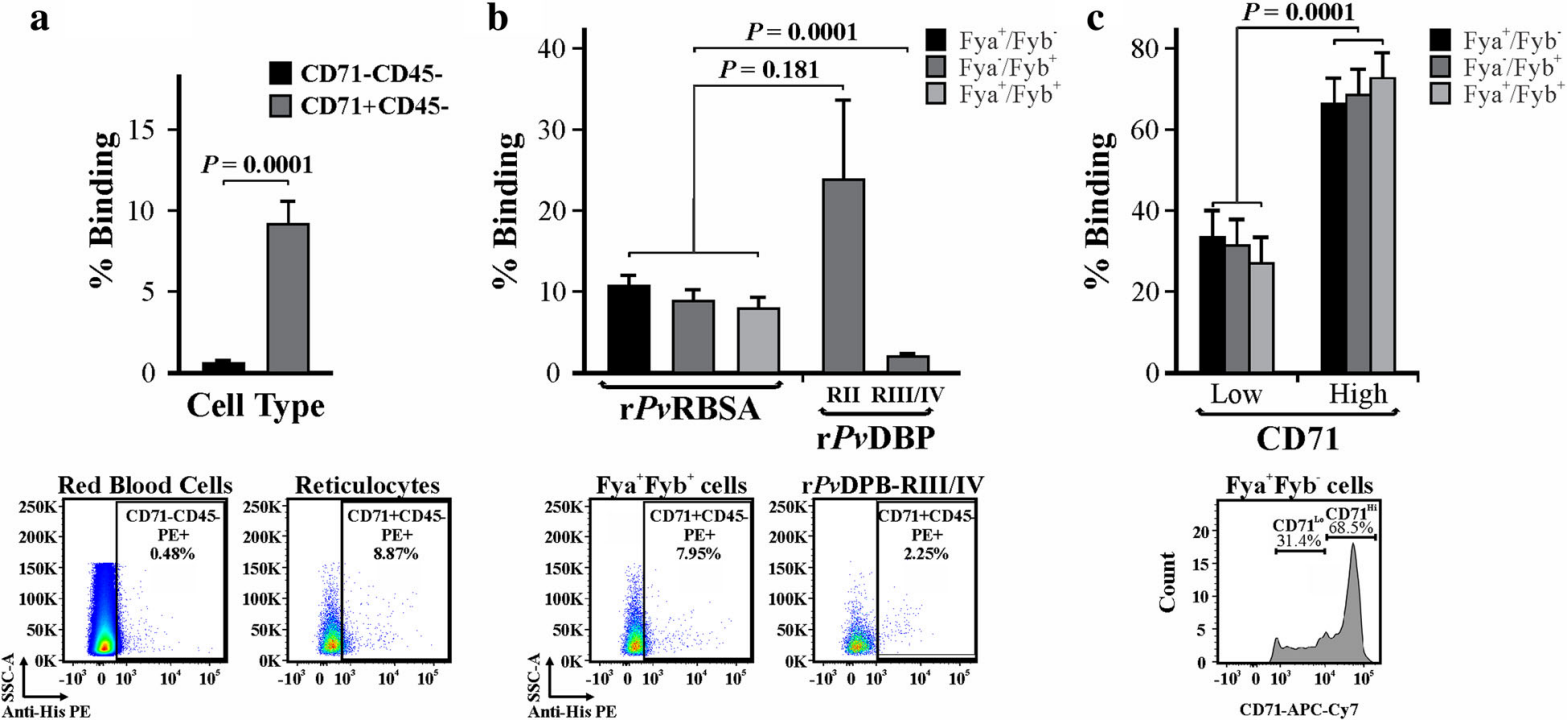
r*Pv*RBSA binding activity to target cells. **a** r*Pv*RBSA binding percentage to erythrocytes (CD71-CD45-) and reticulocytes (CD71 + CD45-). **b** Percentage r*Pv*RBSA binding to reticulocytes with a different Duffy phenotype and **c** regarding CD71-APCH7 signal. A representative dot plot or histogram used for building the bar chart is shown in the bottom part of each figure. Binding percentage in three analyses were expressed as mean ± SD of three independent experiments

Flow cytometry was used for quantifying r*Pv*RBSA ability to bind cord blood reticulocytes using a gating strategy to exclude cell debris and select the CD71 + CD45- cell population (Fig. 3). The recombinant protein had a curve shift when PE signals from r*Pv*RBSA binding assay and control (using CD71 + CD45- cells) were compared in a histogram. r*Pv*RBSA bound to mature erythrocytes to a much lesser extent compared with reticulocytes (t-test: *t*_(4)_ = 13.74, *P* = 0.0001) (Fig. 4a, Table 2). The protein had similar binding activity to cells having a different Duffy phenotype (X̄ ± SD = 9.17 ± 1.4) and to positive control (X̄ ± SD = 23.8 ± 9.8) (ANOVA-Tukey: *F*_(3,5)_ = 2.43, *P =* 0.181), whilst there was a statistically significant difference in r*Pv*RBSA binding activity compared to negative control (X̄ ± SD = 2.0 ± 0.34) (Fig. 4b) (ANOVA-Tukey: *F*_(3,5)_ = 49.53, *P =* 0.0001). Interestingly, r*Pv*RBSA had higher interaction with CD71^hi^ than CD71^lo^ cells (Fig. 4c) (*t*-test: *t*_(4)_ = 16.44, *P* = 0.0001), suggesting that this molecule binds better to the more immature reticulocyte stages.

**Table 2:** r*Pv*RBSA binding percentage to mature and immature erythrocytes. The mean and standard deviation of three independent experiments is shown for each assay

Molecule	Phenotype	% Binding to mature erythrocytes	% Binding to reticulocytes
r*Pv*RBSA	Fya^+^/Fyb^−^	0.47 ± 0.01	10.7 ± 1.29
	Fya^−^/Fyb^+^	0.48 ± 0.22	8.87 ± 0.65
	Fya^+^/Fyb^+^	0.79 ± 0.23	7.95 ± 1.94

## Discussion

*Plasmodium vivax* has several proteins with essential functions in target cell binding and invasion. An important amount of such proteins has recently been identified in *P. vivax* by proteomics analysis which, combined with *in silico* analysis, has led to partly understanding the complex protein machinery used by the parasite and predicting the functions which some parasite proteins may have [5–8]. Exploiting the information available in proteome studies of *P. vivax*, a large-scale analysis was made for predicting protein vaccine candidates, taking into account the parameters described in the methodology. The screening identified *Pv*RBSA, a molecule whose unique homologue is in *P. cynomolgi,* a species which invades reticulocytes and which is taxonomically very close to *P. vivax* [32].

The *in silico* analysis showed that *Pv*RBSA has the characteristics of a good vaccine candidate, as reported for other parasite proteins. Two transmembrane regions were predicted. Transmembrane helices are usually 20 amino acids long, suggesting that the two helices identified for *Pv*RBSA require a very tight loop to both fit into the membrane. Given these findings (predicted by several programmes), it was considered that the region spanning amino acids 332 to 377 is a transmembrane zone, though future investigation is necessary to ascertain their architecture.

In spite of the difficulty involved in basic research regarding *P. vivax*, given the intrinsic characteristics of its biology [1], the *Pv*RBSA was characterised due to adapting the *P. vivax* VCG-I strain in primates [25], which led to sufficient biological material being obtained for developing the experimental assays. The methods used here showed that the *pvrbsa* gene was transcribed and translated for a surface protein in *P. vivax* VCG-I strain mature schizonts (Fig. 1a, d), thereby coinciding with the finding of *Pv*RBSA peptides being detected in the first proteomic study in Colombia of a primate model-adapted *P. vivax* strain [6]. It has been found that parasite transcripts are strictly controlled during the development of the intra-erythrocyte life-cycle [4, 33] and that their codifying products correlate with having a specialised function. For example, more than 50 different *P. falciparum* transcripts having maximum expression during mature stages (>35 h post-invasion) encode proteins that play an important role during cell invasion [34]. The previous statement, added to the results concerning *pvrbsa* presence and expression in *P. vivax* schizonts, suggested that the molecule could have a function during reticulocyte adhesion.

Another important characteristic regarding proteins to be included in a vaccine is that they should be antigenic since it has been seen that an immune response induced during infection is related to naturally-acquired immunity [35, 36]. It was found that *Pv*RBSA could trigger an immune response during natural *P. vivax* malaria infection (Fig. 2), as described for other surface antigens in the *P. vivax* VCG-I strain, such as *Pv*MSP-10 [37], *Pv*12 [38] and *Pv*ARP [30]. Once *Pv*RBSA localisation pattern and ability to trigger an immune response had been determined, it was ascertained whether the protein could bind to the most immature human reticulocytes using anti-CD71 monoclonal antibody (a specific marker for the cells [39]). r*Pv*RBSA was able to interact with the youngest reticulocyte population (CD71^hi^) having different Duffy phenotypes in similar percentages (Fig. 4b). This binding pattern to cells with different Duffy phenotypes has also been reported for DBP [40].

On the other hand, although r*Pv*RBSA was able to bind to mature erythrocytes, its interaction was much greater with reticulocytes (Fig. 4a, Table 2). Such preferential binding to this type of cells has also been observed in other *P. vivax* proteins such as DBP [9], MSP-1 [13], the erythrocyte binding protein (EBP) [12] and some RBPs [11, 41]. In the case of MSP-1, it was initially thought that target cell selection occurred at a later stage when RBPs were secreted. However, further receptor-ligand studies using *Pv*MSP-1-derived 20-mer long peptides have shown that several peptides bind more strongly to reticulocytes than to erythrocytes, suggesting that this protein participates in the pre-selection of *P. vivax* target cells [13]. Furthermore, it has been shown that *Aotus* monkeys vaccinated with MSP-1 recombinant fragments containing reticulocyte-binding peptides have developed protective immunity against *P. vivax* challenge [42].

A recent study assessing five RBPs’ target cell preference has shown the preferential binding to reticulocytes of just one of them (RBP2b). Interestingly, antibodies against RBP2b, acquired during natural *P. vivax* infection, have shown a strong protective effect [41]. These studies highlight the significant role for this type of molecule in interaction with *P. vivax* target cells. According to the results shown here, *Pv*RBSA was localised on parasite surface and displayed a preferential binding profile for the more immature reticulocyte stages. It can thus be suggested that RBPs are not only participating in *P. vivax* preferential binding to reticulocytes (as was initially thought) but that other ligands are also pre-selecting this cell population, such as *Pv*MSP-1, EBP, DBP and now, r*Pv*RBSA.

## Conclusions

This study has described for the first time, an exclusive reticulocyte-infecting *Plasmodium* species molecule’s characterisation and role in binding. The findings highlight that *Pv*RBSA is present in the *P. vivax* VCG-I strain genome, produces a transcript and encodes a protein having a surface location pattern. *Pv*RBSA is antigenic and is an adhesin protein able to bind preferentially to human reticulocytes. Future studies should be undertaken aimed at assessing the protective efficacy induced when immunising with *Pv*RBSA in the *Aotus* monkey experimental model.

## Additional file

Additional file 1: Recombinant *Pv*RBSA purification. Lane 1: the proteins’ molecular marker; Lanes 2–6: eluted protein using buffer with increasing concentration of imidazole (50 mM, 100 mM, 200 mM, 300 mM and 500 mM) stained with Coomassie blue; Lane 7: recognition of r*Pv*RBSA by Western blot using anti-polyhistidine antibodies. (TIF 1539 kb)

## Abbreviations

ANOVA: Analysis of variance.
CD71^hi^: CD71 high
CD71^lo^: CD71 low
cDNA: Complementary DNA
DBP: Duffy binding protein
EBP: Erythrocyte binding protein
ELISA: Enzyme-linked immunosorbent assay
GAMA: GPI-anchored micronemal antigen
gDNA: Genomic DNA
LB: Luria-Bertani
MSP-1: Merozoite surface protein-1
NEB: Native extraction buffer
OD: Optical density
*Pv*RBSA: *P. vivax* reticulocyte binding surface antigen
RBP: Reticulocyte binding protein
RON5: Rhoptry neck protein-5
RT: Room temperature
SD: Standard deviation
VCG-I: Vivax Colombia Guaviare I
