# Supplementary figures and images for: Characterising PvRBSA: an exclusive protein from Plasmodium species infecting reticulocytes
Source: Parasit Vectors. 2017 May 18;10:243. doi: 10.1186/s13071-017-2185-6 (PMC5437689; doi:10.1186/s13071-017-2185-6)

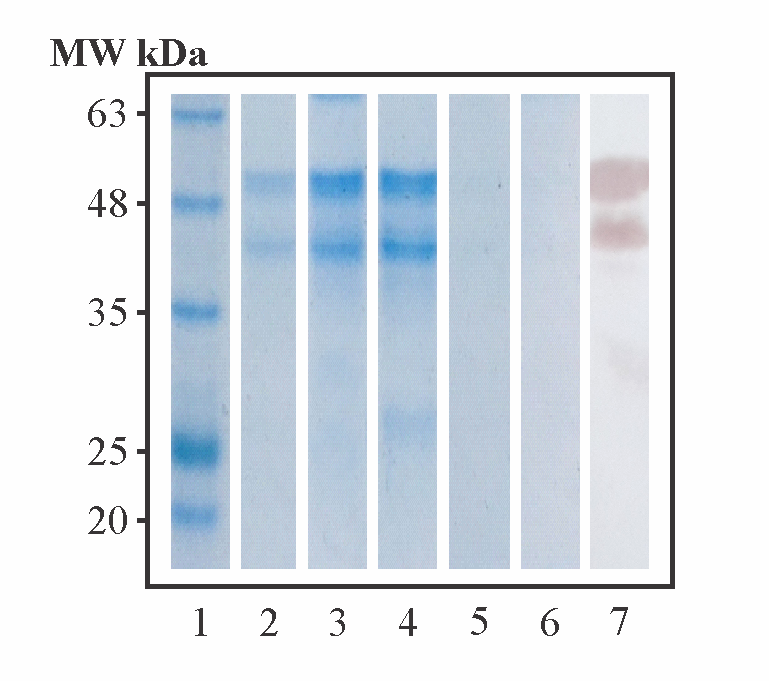

Supplement: Additional file 1: — Recombinant PvRBSA purification. Lane 1: the proteins’ molecular marker; Lanes 2–6: eluted protein using buffer with increasing concentration of imidazole (50 mM, 100 mM, 200 mM, 300 mM and 500 mM) stained with Coomassie blue; Lane 7: recognition of rPvRBSA by Western blot using anti-polyhistidine antibodies. (TIF 1539 kb) [file 13071_2017_2185_MOESM1_ESM.tif]
